# That mr. Alzheimer… you never know what he’s up to, but what about me? A discourse analysis of how Swedish spouse caregivers can make their subject positions understandable and meaningful

**DOI:** 10.1080/17482631.2018.1554025

**Published:** 2018-12-12

**Authors:** Annica Lövenmark, Martina Summer Meranius, Lena Marmstål Hammar

**Affiliations:** a School of Health, Care and Social Welfare, Mälardalen University, Västerås, Sweden; b School of Education, Health and Society, Dalarna University, Falun, Sweden; c Division of Nursing, Department of Neurobiology, Care Sciences, Karolinska Institute, Stockholm, Sweden

**Keywords:** Alzheimer’s, caregiving, discourse analysis, dementia, experience, interviews, subject positions, spouses, qualitative

## Abstract

The spouses of people suffering from dementia are commonly first-in-line caregivers. This can have a considerable effect on their own lives, health and marriages. Several studies have focused on spouses’ experiences, but very few have focused in any depth on their descriptions of themselves as subjects. Therefore, the aim of this study is to describe how spouse caregivers can express themselves when living with and caring for their partners with dementia. The study has a qualitative approach with a discourse analysis design and uses analytical tools such as rhetoric, subject positions and categorization. The results reveal three subject positions: as an actor, as a parent and as a survivor. The results show that as spouses struggle with external and internal clashes as subjects, they therefore need to develop coping strategies. They also experience pronounced loneliness and a risk to their own health. There is thus a need to support these spouses as individuals in their differing and changing needs.

## Introduction

Globally, it is estimated that 46.8 billion people live with dementia and that this number is growing rapidly due to an increasingly older population. It is estimated that in 2030 the figure will be around 75 million and by 2050, 132 million, which makes it one of the world’s most serious public health challenges (Association, 2016; World Health Organization [WHO], 2017). Dementia results in an extensive range of disabilities, such as a decline in or loss of memory and language, disorientation and an inability to plan, as well as behavioural symptoms such as aggressiveness and resistance. As these disabilities and symptoms increase in severity as the condition progresses, the person suffering from dementia often requires 24-h supervision (Cerejeira, Lagarto, & Mukaetova-Ladinska, 2012). Importantly, dementia does not only affect the person with the condition, but also the quality of life of family members (Ask et al., 2014; Eloniemi-Sulkava et al., 2002; Johannessen, Helvik, Engedal, & Thorsen, 2017).

When those with dementia live at home, spouses are often the first-in-line caregivers. This often has a severe impact on their life and their health. Studies on the experiences of spousal caregivers have shown that they have a perceived lower quality of life than the person they are caring for (Adams, 2008; Ask et al., 2014; Balducci et al., 2008; Wadham, Simpson, Rust, & Murray, 2016). In their review of research on spouse caregivers of people with dementia, La Fontaine and Oyebode (2014) conclude that the relationship between the partners is often strained, and that the caregiver can feel trapped, lonely, lack support and have little time for themselves in the caregiving situation. In addition, they report feelings of losing their partners due to difficulties such as sharing thoughts, feelings and experiences as a couple, which in turns leads to reduced intimacy and a sense that they are no longer married (Ask et al., 2014; Eloniemi-Sulkava et al., 2002; Kaplan, 2001; Pozzebon, Douglas, & Ames, 2016). Tumola, Soon, Fisher, and Yap (2016) highlight that spouses experience not having enough time for themselves, that they bear a heavy burden due to not having enough rest, feelings of guilt, the loss of a sense of self and having to accept their fate. There is also the possibility of historical artefacts in the form of negative stereotypes or associations of mental health, dementia and institutional care, all of which produce fear and stigma and contribute to keeping dementia a hidden condition (Stokes, Cobes, & Stokes, 2014). There are also positive sides to caring for someone with dementia. For example, caring for a partner is regarded as a cultural right and a good thing to do (Han & Radel, 2016; Shim, Barroso, & Davis, 2011). In their study, Merrick, Camic, and O´Shaughnessy (2016) found that both the person with dementia and the spouse are motivated to maintain their “couplehood” relationship (as described by Hellstrom, Nolan, & Lundh, 2005) and the dementia patient’s personhood. However, a loss-oriented health and social care that does not adequately support these couples can contribute to a loss of personhood for those suffering from dementia (Merrick et al., 2016). Studies also show that caregivers devise strategies in order to maintain the couplehood. Couples affected by dementia should be viewed as a unit, rather than two separate individuals, and devise strategies based on their couplehood to maintain the relation, by for example engaging in the relationship, doing things together (Han & Radel, 2016; Hellstrom, Nolan, & Lundh, 2007), having good communications (Williams, 2015; Williams, Newman, et al., 2017a), working things through together and moving on and letting go (Hellstrom et al., 2007). Strategies for maintaining good relations are also highlighted in previous research (e.g., Hernandez, Spencer, Ingersoll-Dayton, Faber, & Ewert, 2017; Myhre, Bjornstad Tonga, Ulstein, Hoye, & Kvaal, 2017; Riley, Evans, & Oyebode, 2018). Myhre (2017has focused on coping strategies for caregiver spouses and the need for them to focus on themselves as subjects in the relation.

As described, several studies have focused on the experience of being a caregiver spouse to a person with dementia and their strategies. Up to now, data collections have mainly revolved around spouses’ expressions of their own experiences and perceptions. In their descriptions, they often refer to themselves as subjects. As far as we are aware, no studies have been conducted on caregiver spouses of people suffering from dementia and how they express themselves as subjects in the relation in a conversation. This is a way of exemplifying and more carefully explaining their experiences, which in turns yields valuable information and helps us to fully understand the spouse’s situation when developing support interventions for this particular group and hopefully strengthen their ability to experience health and well-being. Therefore, the aim of this study is to describe how caregiver spouses can construct themselves as subjects when living with and caring for their spouse with dementia.

## Theoretical framework

Discourse is the logic and structure of conversation that determines the limits of what is socially and culturally sanctioned and can be sanctioned (Foucault, 1971/1993). Discourse regulates which expressions are accepted or not in certain situations or contexts. Language is a tool in an ongoing construction of ourselves and the world and determines what is possible to say, do and feel (Willig, 2008). Different forms of speech constitute instruments for and expressions of the ways in which power, individuals and the physical world are staged (Howarth, 2007). Language is an act that shows how realities are staged and invested with value and content. It is also a social practice in the sense that language is creative, makes something, delimits, performs, stages and reproduces thoughts, actions and what we see as true, right and important. Language governs what we talk about and how we talk about it (Hall, 2001).

## Aim

The aim of this study is to describe how caregiver spouses can construct themselves as subjects when living with and caring for their spouse with dementia.

## Methodology

This study has a qualitative approach with uses discourse analysis as a design and analysis method. The state of being a spouse is socially constructed, in that it is a social arrangement that we often take for granted as something ´natural´. This because it is based on our expectations and values about what is common and natural for a married couple and when one of the partners falls ill (e.g., “for better or worse”), even if the features and content of being a spouse have varied culturally and over time. It is also a social category and the starting point for an analytic discursive perspective (Potter & Wetherell, 2001).

Subject position is a concept of identity and the self (Edley, 2001) that is made possible through language. A subject position becomes possible and is constructed from what an individual perceives to be most meaningful, logical and suitable in a certain situation or specific context (Hall, 2001). The self is ´made´ in interaction with other subjects, and different subjects become relevant through specific ways of using language (Edley, 2001). The identities of different groups are constructed and made visible in their discursive and rhetorical contexts (Billig, 2001).

In this article, the analytical focus is on how spouses use categorization to organize, systematize and make their conceptions of reality possible and meaningful and to create a common understanding of them as subjects. As a rhetorical resource, categorization has an argumentative function. The analytical focus is on how they “do subject” by occupying subject positions that are negotiated on the basis of whatever is in focus in a certain context. Some categories entitle people to certain actions and knowledge, for example doctors or teachers, and sanction a certain legitimacy and credibility (Potter, 1996). Categorization is part of the social order and resources in our construction of relationships and situations in daily life and is used to understand, negotiate and establish an identity as a spouse. The analytical focus on categorization shows how spouses negotiate their subject positions as possible, enforced or meaningful. The explicit categories that are created by the spouses (e.g., actor, attachment Table AI) and the analytically formulated categories (e.g., supervisor, attachment Table AII) are abstracted into analytically formed subject positions (e.g., parent or survivor, attachment Tables AII and AIII). The analytical tools used by Seymour-Smith, Wetherell and Phoenix show how categories construct subject positions when doctors and nurses talk about female and male patients (Seymour-Smith, Wetherell, & Phoenix, 2002). The category “men” is treated in the same way as the category “child”, e.g., in talk about men in need of being fostered and the categorized actions of kicking and screaming. This is exemplified as a man as a recalcitrant child, constructed on the basis of his female partner telling him to act and behave well. Here, the normative characteristic of a category is transferred from one category (child) to another (man) in an attempt to understand and sanction the man’s subject position as an irresponsible child regarding his own health and the female subject position as a supervisor with moral responsibility (Seymour-Smith, Wetherell, & Phoenix, 2002). Reynolds and Wetherell (2003) analyze how individuals negotiate their belonging to a specific category and how it should be defined. For example, this is acted out by recruiting the category “married” when talking about the possibility of getting married but choosing to stay single. In this way, the category “single” is constructed as less problematic and the single person is not regarded as insufficient or impossible in a partnership (ibid.)

### Setting and data collection

Nine spouses whose partners suffered from dementia took part in the study. The couples lived in their own homes in three medium-sized cities in an urban area of Sweden. Of these nine spouses, four were men and five were women, all of whom were between the ages of 65 and 94 and had been caring for their partners for the last 2 to 8 years in their own homes. Three of the participants were asked by their spouses’ physicians at the memory clinic they attended whether they would be willing to participate in the study. The rest were asked to participate by the convener of a support group from their local dementia federation. The spouses were interviewed by a member of the research team in their homes. The interviews were semi-structured (Polit & Beck, 2016) and focused on their relationships and lives as a couple affected by dementia. Each interview lasted for about 1 h. None of the participants had prior experience of or training in caring for people with dementia.

The situations and contexts include the specific storyteller context (Potter, 1996) in which the spouse and the researcher interact, and the spouses’ stories about their daily lives. The analysis highlights the different ways of talking about themselves as spouses and how they negotiate, show and occupy a subject position (actor, parent or survivor). The result shows the different categories the spouses create (e.g., supervisor, facilitator, parent) and the categorized actions (e.g., governing, supervising, facilitating, nagging) that are used in the construction of a meaningful, suitable and logical subject position. The storylines of everyday conversations and interactions with their partners and with other people (e.g., the researcher, other caregivers etc.) provide a position from which to speak and facilitate the positioning of others as characters with roles and rights. Who the spouse can be depends on the positions that are made available through talks, interactions and conversations. The analysis shows which subject positions the spouses negotiate, take for granted or are the only ones possible in their particular situations and contexts.

### Analysis

The analysis aimed to identify and schedule the participant categorized action and the participant person categories that were manifested and became visible in the interview texts. The first step was to identify the categorized actions and person categories that were not explicit in the text, but which could be abstracted as researchers’ categorized actions and researchers person categories. All forms of categorized actions and persons were organized into separate groups based on common features and content. Thereafter, all the person categories were combined with the categorized actions that supported them in some way. The next and last step was to abstract all the person categories into subject positions.

The empirical examples that are presented and analyzed in this article illustrate how spouses use language to negotiate belonging to subject positions that make the person categories and actions possible, logical and meaningful. The Tables (AI–AIII) in the appendix show all the category actions, person categories and subject positions that were identified in the analysis and how they relate to each other: the *subject position as actor* (attachment Table AI), the *subject position as parent* (attachment Table AII) and the *subject position as survivor* (attachment Table AIII). The result shows the categorized actions and person categories that build the subject positions.

### Ethical considerations

The Regional Board of Research Ethics (record number: 2016/446) reviewed and approved the study. The World Medical Association Declaration of Helsinki was carefully considered. Prior to the data collection, the participants were provided with oral and written information about the study, including its voluntary nature and that they could withdraw at any time. The participants provided signed informed consent before the interviews took place and were guaranteed confidentiality. All the names presented in the results section are fictitious.

## Results

Living with and caring for a partner who has been diagnosed as having dementia required the spouse to enter a whole new world and perform new roles (subject positions) that were difficult to imagine, rehearse or prepare for. A common theme for the spouses in this study was the constant shift between subject positions in order to manage daily life together with their partners. The circumstances forced them to be aware, start out from and responsively return to a subject position as a flexible and skilled actor, from which they could take on any role (subject position) that was necessary or possible in the situation and context.

The situations and contexts that the caregiver spouses talked about also conditioned and governed the stories they told. In their homes the spouses often appeared as unaware and constantly improvising counter-actors, i.e., they could turn their hands to anything and adopt any role anytime and anywhere. With time, the spouses developed some of the skills that were necessary to manage daily life with a partner suffering from dementia. But there was a great price to pay, for example in terms of the loss of self, a life of their own, former companionship and known roles such as wife/husband etc. They became full-time actors and, for the play to end, the spouse or partner had to leave the common stage permanently, in some way or other.

### The spouse as actor

Albert (IP 1) and his wife were married 32 years ago and Maj had been ill for the past 7 years. Albert regarded himself as an actor: ´…my children asked me—how can you keep a poker face… Yes, maybe I am a bit like an actor…´ (IP1). Albert occupied the subject position of *actor* by saying that his children asked him *how it was possible* for him to *keep a poker face*. Maj and Albert still communicated physically through kisses and hugs, and Maj enjoyed patting Albert´s head:

…she can stand behind me and pat my head. I really don’t like it. I tell her ´- I´m not a dog Maj´, because we used to have a dog, but nowadays I don’t say anything, I let her pat… (IP 1)

Albert negotiated his belonging to the subject position of actor with the categories *actor* and *dog*. In order to play along, Albert *accepted the false reality* and momentarily *denied* his identity as a human being and instead *acted* like a *dog*. Albert could therefore be the *skilled actor* that had *given up* trying to say anything that might *change the role he had been assigned*, which by *experience* he knew would not work.

Albert was not alone in using actions that supported the subject position of actor. Siv (IP 6) lived with her husband Ulf who had been ill for 6 years and struggled to keep a poker face in her actions: ´…I hold it inside myself, or go the bathroom one more time or… I can´t leave him alone anymore and go outside. So… no, but I actually have to bite my tongue and pretend…´ (IP6). In the telling context, Siv negotiated and stated that she was an *actor* because she could *hold it inside, bite her tongue, hide* and *pretend*. Another statement that supported this subject position was that she *could not tell the truth*:

…He´s afraid of losing me, or that something might happen to me… so I avoid telling or talking about how I feel. Because he… if I tell him that I´m tired, ´Yes, but why are you tired, you slept last night?´ I can reply, but we have been awake a bit. The he says ´Yes, but so have I and I´m not tired.´ So he doesn’t have that understanding anymore, that you can be tired. (IP 6)

Siv was both *wanted* and *experienced* as a *skilled actor* because she claimed that her partner was afraid of losing her, or that something might happen to her. She argued for the *necessity* of the belonging and for appearing as a *skilled actor* by saying that she *could not tell the truth* because her partner was no longer able to understand her feelings or needs. As a result, she *hid* her negative feelings in order to keep him calm or happy. Thus, as a skilled actor, she tried to *avoid* ending up in a hopeless discussion.

Having to hide the truth or even lie was described as something awful and difficult for Anna (IP 5), who lived with her husband Mike, who suffered from dementia:

…yes… and then it´s just that thing about these white lies that I tell, I think it´s awful, because I have never lied to my husband before. But now I have to do it. And I think it´s very hard. (IP 5).

As a *skilled actor* Anna argued that *lying* was *necessary*. She stated that she *had* to do it and also claimed that lying was what we would all do if we were in her situation. In this way she was able to negotiate the fact that she was a *skilled actor*, was not a liar and did not lie to others.

### The spouse as parent

Albert (IP 1) described parenting as a spouse like this:

The homecare was here to help with her personal hygiene… and she´s asleep when they arrive, so I´m up by then and stand beside to help them…//…and say, here are her clothes and here is this and that…//…and then I used to shower her genitals every day, because urine has a very strong smell…//…so I smell for her… she has no sense of smell anymore…it’s dead… one thing after another stops functioning…//… I´m used to this now… (IP 1)

Albert created the subject position of spouse as parent with the characteristic actions of a *facilitator* who *facilitated* for the homecare professionals or his wife. The *parent* is also constructed when he had to be *the good one* and *be up* and *be there for them*, to be the *expert* and *tell* and *show* where things were. He had to be the *parent that´s up* and, the parent that *showered, smelled* when there was a need for clean clothes and *know what´s needed because functions she needs are not there*. Albert was also the *parent* by being *the good one* who was *used to* doing that day in and day out and was *sustainable* and *sustaining*.

Ulf and Rosa (IP 8) had lived together for over 50 years. Ulf had been ill for many years and needed a lot of *parenting*:

…I use to say that he´s like a four-year-old child…I say ´Have you brushed your teeth?´,- ´Yes I have´… ´Yes but Ulf, you haven’t´.,—´Yes I have´…—´No, but I know you haven´t, I´m here with you in the mornings and at night, you haven´t done it´,—´Well then I haven´t” he replies a little bit angry and then goes off to brush his teeth´… (IP 8)

Rosa was the *expert* who was *needed*. She *asked, explained, cajoled* and *argued* with Ulf about the fact that he had not brushed his teeth and that this was *necessary*. She was the *supervisor* who *nagged* and as a *mother* compared Ulf with a small *child*. As the *good one* she was *firm but not bossy* in her *mothering*. As the *expert*, she gave the impression that she was the kind of spouse who was a *good parent*. That made it possible for her to draw on a common understanding and taken for granted discourse about what is characteristic for a good and *qualified parent*. With her telling, she mediated that she could be the good one because she *cared, knew when to be quiet or not, was there* and was *patient, calm, active* and *self-controlled*. As *parent* and a *constant worker* she was *never off duty* because she was all the time *shadowing, supvervising, governing* and *protecting*. As a *parent* she´s also *the good one* when being an *expert*, she was *competent* and *remembered*.

Maud (IP 2) had lived with her husband Henrik for over 55 years. Henrik had been ill for 2 years. When Maud talked about their daily life Henrik was in the room, sitting on the couch chewing pieces of newsprint. Maud explained: ´…I think he´ll be finished soon…now that I’ve allowed him to eat that…I don’t care if he does… It won’t kill him…´ (IP 2). Maud´s actions as a spouse as *parent* were to be an *expert* and *explain* by indirectly pointing out that she was the one who only *picked the fights that were worth dealing with* (“Now that I’ve allowed him to eat that”). In this sense she was actually *the good one*, because she was *caring, flexible* and could *endure* (“He´ll be finished soon”). She was also an *expert*, in that she was able to *evaluate* odd behaviour as something that was not dangerous or lethal (“It won’t kill him”). She was the kind of spouse as parent who was a *mother* because she did the *caring*, a *facilitator* who *facilitated* and a *supervisor* who *supervised*. She had also stopped *nagging* him about certain things and *allowed* him to do them. Therefore, she was also *the good one* because she was *patient* and *protective*. At the same time, she was *the constant worker* who was *never off duty*, but was *tired* of trying to make him stop eating newsprint. Belonging to more than one category at once, or being stuck in one of them, made her in some way *trapped between her own needs and the needs of her partner*. The challenge was to let him do want he wanted, or to be the *parent* that can be a *facilitator, supervisor* and *constant worker* and at the same time an *expert* and *mother* who struggled with a *child* who did not have the capacity to understand what was wrong or why.

### The spouse as survivor

Anna (IP 5) talked about the subject position of a spouse as a *survivor*:

…It´s hard… now I start to cry, because I don´t have… I can´t talk to him as I want to, because I don’t get an answer…//…I miss him, I don’t have the man I married today…//I’d rather talk to the dog than him, I get more answers from her than from him today…//…It´s pointless to say anything [about how I feel]… because… yes, often… then he only says “but send be to the care home then´… If he´s in a good mood… and understands what I´m saying… “send me to the home, like they did with my mum…´//… then he´s like…moody… I´m not allowed to feel bad, no, it´s just so, I´m not allowed to feel bad because then he gets moody. That’s how it is. I´m just supposed to shut up, sit beside him, then it´s at its best… (IP 5)

Anna started to cry when she talked about what was required of her in trying to *survive*, she has to be the *strong one*: she *grieved* because she *missed* the man she once married. He had *gone* and she was *lonely*, despite the fact that they were still living together. He was there in a physical sense, but there was *no longer any point* in talking to him. Saying that she would rather talk to the dog supported her sentiments of how *pointless* it all was, because a dog cannot talk in the way that humans can. She needed to be the *strong* and *healthy* one and *hide* the fact that she did not feel good, because if she felt bad and showed it, her husband became moody and said that she *could get rid of him* by sending him away. This created a loser situation for Anna. She was the one who had to *feel guilty* about not being content or happy, or showing it, and being the one who could or wanted to send him away. She had to *contain* and *hide* her own *needs* and *feelings, survive* by being the *strong* person, but also *be the one who did not exist*. Being ill or needy as a spouse in this situation was not possible. She had to *survive* and stay *strong* and *healthy* and to *accept* that *that was how things were*. Siv (IP 6) also highlighted the *loneliness* and lack of professional support when she talked about herself as a *survivor on her own* in a *new world*: ´…it´s a whole new world to me this…//… you´re very lonely…´ Siv was the one who *did not exist* and who had to stay *healthy*, feel *guilty* and *survive* against all the odds:

…my life has disappeared… and now everything is gone…It decreases that I am his wife…//…you´re very lonely at home… I´m a bit down and depressed so I think I have to go out and walk, get some fresh air and new energy… I have to survive this so I have to do it then… I feel ashamed, like I´ve caused it… (IP 6)

Being *healthier* and the *survivor* and knowing that created a feeling of *guilt* (“I feel ashamed”). It made Siv wonder whether she was the *cause of the disaster*.

Rosa IP (8) had cancer, but as a survivor spouse had to focus on her husband:

…We are two, but I´m alone…//…it´s strange…I´m frustrated…and in some way you have to deal with that…//…I have metastases in my back so I can’t run, but I can walk…//… I have treatment every third week… cytostatic, I´m not supposed to lift him up when needed, but I do it anyway… this is my new assignment… (IP 8)

As a *survivor* (in her new assignment) Rosa was *alone* in the relationship and thought that it was both strange and frustrating to live like this, but she knew that there was *no point looking back* (“You have to deal with that”). Rosa was very ill and had regular cytostatic treatment. Even though she was not allowed to do any heavy lifting, she prioritized her husband’s needs and comfort before her own (“I´m not supposed to lift him up when needed, but I do it anyway”). As a *survivor* spouse she had to be *strong* and act as though she was *healthier* than her husband.

Living with a *stranger* was one thing, but living with someone who was drifting away from you could be frightening:

… You know… I get scared when you talk like that…when you say that you are going to beat me to death… ´Yes, but you know´, he says, ´you know that I never will do that…´ No, I say… not You Ulf, but that Mr. Alzheimer, you never know what he will get up to… And then he says ´Yes, that’s obvious… because you really can’t know…´ (IP 6)

Siv (IP 6) said that her husband *scared* her when he threatened to beat her up. Her husband argued that she *should know* that he was not that kind of person. Siv told him that she *did not know that anymore* because Ulf no longer existed and she was now living with Mr. Alzheimer, and *she did not know him at all*. She explained that Mr. Alzheimer was not to be *trusted*, because you *never knew what he was up to*. Sivs´ husband managed, at that moment, to in some way capture the fact that he was both Ulf and Mr. Alzheimer and admitted that is *how it was*. She´s the only one that *survived* the disaster and is left among other *strangers*. The survivor had to survive and be the one that’s strong and healthy and at the same time cope being a stranger and the one that in some sense doesn´t exist anymore, because the disaster hit them.

## Discussion

This study describes the subject positions of spouses caring for partners suffering from dementia. The results reveal the construction of several subjects. Together they express being the ones who steer the situation, help the couple to move forward in their lives together and devise coping strategies. As Ask et al. (2014), Eloniemi-Sulkava et al. (2002) and Johannessen et al. (2017) suggest, a dementia condition affects the quality of life of family members over a number of years. Spouses need to find coping strategies to maintain their own health and marriage, and at the same time be a caregiver. Williams (2011) found that spouses with strong relations are less likely to be depressed or feel overburdened. It could thereby be argued that there is a need for extended support in order to maintain the relationship. Williams (2015) suggests that it is important to relate to the other with compassion and to trust in the existence of the attachment of the marriage, and that these aspects should be articulated in the communication (Williams, 2015; Williams, Newman, et al., 2017b; Williams & Parker, 2012b). However, as in any relation, taking care for yourself is important, which can be problematic when living with a spouse with dementia. The results reveal that these actors are never off duty, need to have or learn certain skills and have to constantly tend to their partner’s needs, regardless of whom they want or need to be themselves. This can be linked to studies about being lonely in the situation and the lack of support for or possibility of taking care of themselves (La Fontaine & Oyebode,2014; Eloniemi-Sulkava et al., 2002; Kaplan, 2001; Pozzebon et al., 2016). As well as as describing themselves as parents, they no longer experience themselves as being married; an aspect that is also supported in previous research (Pozzebon et al., 2016). The spouses have to switch between necessary subject positions in order to support their partners, family or other caregivers. This study also supports the findings of Tumola et al. (2016), namely that spouses lack time for themselves, that they are obliged to accept their fate and have to try to stay healthy. The burdens that Tumola et al describe are also present in the form of carrying the weight of being a parent and coping with the loss of both themselves and their partners.

However, this study also shows that the challenge for these spouses is not only having to cope in the situations they are confronted with, but that there is an external and internal struggle for them as subjects, in that they have to manage the clashes between and within their own subject positions, or lack of them. The clash for the spouses as actors is to drop the mask in order to be true to themselves and at the same time stay in the assigned role in order to make life easier for their partners. The clash for the spouses as parents is to prioritise their own needs and well-being and at the same time take account of the needs and comfort of their partners. The clash for the spouses as survivors is to think of their own survival and that of their partners so that the latter can stay at home for as long as is possible.

Being a spouse is also a kind of stigma. Stokes et al. (2014) highlight the fact that stereotypes have consequences for people with dementia, but this study shows that this is also the case for the spouses. It could therefore be said that couplehood (Hellstrom et al., 2007; Hernandez et al., 2017; Myhre et al., 2017; Riley et al., 2018) or the focus on coping strategies (Myhre et al., 2017) could in some cases be quite devastating, in that not only is more weight put on the spouses’ shoulders, but that the clash between subject positions can be fuelled and the fragile boundaries left for the spouses as own important subjects erased. This in turn can also affect the spouses’ own health. As Merrick et al. (2016) state, a loss-oriented health and social care can lead to the loss of personhood for those suffering from dementia and for their spouses.

Knowledge about the kind of challenges that spouses are faced with and the kind of support they can expect is therefore important. The situations of spouses differ depending on which culture they belong to (Han & Radel, 2016). Also, as yet we know very little about what kind of challenges future generations will be confronted with. What will future spouses find acceptable? Will they stay with their partner at the cost of their own health? Will they be willing to sacrifice themselves and their lives? With an increase in mental health issues even amongst the younger generation, what kind of measures will need to be put in place already now to help caregiver spouses?

### Strengths and limitations

The discursive approach is a strength in the sense of the methodological contribution it makes to this field of research. As both a strength and a limitation, the empirical material is constructed in a specific research context and in terms of participants’ interactions with the researcher. It is important to remember that the spouses are placed in specific situations and may want to be perceived in certain ways (Edley, 2001). On the one hand, the researcher is someone with authority due to occupation and the organized situation. On the other hand, the participants have a specific and legitimate position from which to speak. Therefore, even though the researcher may have certain powers regarding the situation and the questions posed, the participants have power over what and how to describe and validate themselves as spouses. There are different ways of talking about yourself as a spouse, e.g., by negotiating, showing and occupying a subject position (Reynolds & Wetherell, 2003). Even though this study is limited in terms of the number of interviews conducted, the result clearly contributes to supporting and challenging previous research. The empirical examples presented and analyzed in this study illustrate different ways of how spouses can use their language to formulate subject positions and their actions in order to organize, systematize and make their conceptions of reality possible and meaningful. By their use of rhetorical resources and what these achieve and convey, it is their version of daily life and how they want others to perceive them as subjects that come across (Hall, 2001; Potter, 1996). According to Guba and Lincoln (1989), there are certain markers for trustworthiness, such as credibility, dependability, conformability and transferability. Credibility is regarded as being thorough in the data collection and analysis. In this study, one researcher performed the interviews and all researchers evaluated the analysis process and results. In the method section, the research- and analysis process is described and easy to follow, so that it is clear that the criterion for dependability is fulfilled. The criterion of conformability is respected through the interview extracts that show that the result is grounded in the collected data. Transferability is fulfilled through the discussion of the results.

## Conclusion

It is clear that caregiver spouses need to have a range of skills in order to occupy immediate, possible, necessary and meaningful subject positions. They also try to manage their daily lives by switching between subject positions. One way of trying to understand the meaningful and possible self as a spouse in daily life is to occupy the subject position of survivor when the positions of actor or parent are not sufficient. This is based on the fact that they are strangers to their partners, and vice versa. This will always be the case, because it is impossible to predict what might happen. It is therefore difficult to know who they need to be or can be. They have to keep on surviving, try to live in the moment, always be aware and be the skilled actor that also can slip into parenting mode when needed.

The result shows that spouses have to struggle with both external and internal clashes for themselves as subjects. When any one subject position is not sufficient, they are faced with a single option, namely to adopt the subject position of survivor for both themselves and their partner. There is an increased risk that this will only require more intense coping strategies, pronounced loneliness and health risks. There is a need to support these spouses as individuals with different interventions, such as training that will help them to care for their spouse in tried and tested ways, as the intervention by (Williams & Parker, 2012b; Williams, Newman, et al., 2017a; Williams, Newman et al., 2017b). In any further research, it would be interesting to analyze which subject positions spouses find possible and meaningful after having received that kind of support.

Finally, the discursive psychology approach used in the study not only supports and contributes to previous research, but also adds to the healthcare and political discourse about how to manage welfare and public health in society today and in the future. Both the person who is diagnosed with dementia and the healthcare system are highly dependent on the fact that spouses exist and willingly take on and endure the assignment of caregivers

## Appendix

**Table AI.** Subject position spouse as actor, person categories and category actions.

Subject position	Spouse as actor		
Person Categories Participants,	*Actor*		*Dog*
Person Categories Researcher,	Actor	Skilled actor	Dog
Participants Categorized Actions	“*keep a poker face”, “a bit like an actor”, “hold it inside”, “be able to lie”, “pat my head”. “I´m not a dog”, “I let her pat”, “we used to have a dog”, “, “to bite together and pretend”, “I avoid telling or talking about how I feel”, “he doesn’t have that understanding anymore”, “ “it´s just one of the white lies”, “I want to be here beside you. “*	*“I don’t say anything, I let her pat…”, “…He´s afraid of losing me or that something could happen to me”, “I avoid telling or talking about how I feel”, “these white lies that I tell” “I have never lied to my husband before”, “you have to do it”, “*	*“Pat my head”, “I´m not a dog”, “I let her pat”, “we used to have a dog”*
Researchers Categorized Actions	acting, hide, pretend, deny the truth, can´t or doesn’t tell the truth, accept the false reality for the moment	wanted, experienced, flexible, know when something is important/necessary, the assigned role doesn’t defines the original self protects, helps avoiding unwished or uncomfortable consequences or situations, has given up on trying to change an assigned role	accept false reality for a moment, deny his real belonging, acting like a dog

**Table AII.** Subject position spouse as parent, person categories and category actions.

Subject position	Spouse as parent					
Person Categories Participants						
Person Categories Researcher	Mother	Facilitator	Expert	Supervisor	The good one	Constantly worker
Participants Categorized Actions	*“I use to shower her”*, “*have a child”*, “*shower”, “so I smell”, “now I allow him to eat that…I don’t care if he does… He doesn’t die from it”*	*“stand beside to help them”, “now I allow him”,*	“*and tell and show”, “have you brushed your teeth’s?”, ”but you haven´t”, “No, but I know you haven´t”, “now I allow him to eat that”, “He doesn’t die from it“*	*“I´m here with you in the mornings and the nights”, have you brushed your teeth’s?”, ”but you haven´t”, “No, but I know you haven´t*” *“you haven´t done it*”, *“now I allow him”*	*“be up…and stand beside to help them‘- Have you brushed your teeth’s?’, ‘now I allow him to eat that’, ”I think he´s finished soon”,*	*“be up”*, “*used to”, “every day”, tired*”, ” *I´m here with you in the mornings and the nights”, “now I allow him to eat that”,*
Researchers Categorized Actions	mothering, care, parenting, to know about her needs and see to that she got the daily care she needs	show, facilitate for your partner	can tell, listen, show, ask, explain, remember, ponder, interpret, valuate, argue, understand, solve problems, know what´s necessary, know when needed, only pick the fights worth dealing with, pick fights needed, qualified parenting, have knowledge, know about certain facts, competent, know what good parenting is	nag, supervise, govern, allow, protect	be there, are there, sustains, loyal, firm but not bossy, care, know when to be quiet, kind, patient, self-controlled, active, adjustable, flexible, inclusive, able to endure, trapped between own needs or wellbeing and others, able to put someone else’s needs or wellbeing in the front, handle remorse	never off duty, shadow your partner, tired

**Table AIII.** Subject position spouse as survivor, person categories and category actions.

Subject position	Spouse as survivor			
Person Categories Participants				
Person Categories Researcher	the strong one	the healthy one	the stranger	the one that doesn’t exist.
Participants Categorized Actions	*“It´s hard”, “now I start to cry, because I don´t have”, “I can´t talk to him like I would, because you doesn’t get any answer”, “I miss him, I don’t have the man I married today”, “I rather talk to the dog than him”, “It´s pointless to say anything”, “but send me to the care-home then”, I´m not allowed to feel bad”, “it´s just so”, “supposed to shut up”, “sit beside him”, I´m not allowed to feel bad because then he becomes moody”, I have to survive this so I have to do it then”, “I feel ashamed”, “like I´ve caused it”, “ “I´m frustrated”, “in some way you have to deal with that”, “I get scared when you talk like that”, “beat me to death”,*	*“I´m not allowed to feel bad”, “it´s just so”, “I´m a bit down and depressed so I think I have to go out and walk, get some pulse and endorphins”, “I have to survive this so I have to do it then”, “I have metastases in my back so I can’t run, by I can walk”, “I have treatment every third week… cytostatic”, “I´m not supposed to lift him up when needed, but I do it anyway”*	*“I can´t talk to him like I would, because you doesn’t get any answer”, I miss him, I don’t have the man I married today…, “I rather talk to the dog than him”, it´s a whole new world to me this”, “you´re very lonely at home, “he´s not my husband”, “I miss having a man”, “I have some dreams of having a life”, “it´s strange”, “this is my new assignment”, ““but that Mr. Alzheimer, you never know what he will get up to”*	*I can´t talk to him like I would, because you doesn’t get any answer”, “I miss him, I don’t have the man I married today “, “I rather talk to the dog than him, “It´s pointless to say anything”, “I´m not allowed to feel bad, “ it´s just so, “supposed to shut up”, “my life has disappeared”, “and now everything is gone”, “you´re very lonely at home”*
Researchers Categorized Actions	try to carry grief and guilt, missing someone, lonely, alone, there´s not point, it´s just like it is, try to hide/contain when they aren’t feeling good/having needs of their own, have less needs than their partner, try to accept that they are the loosing part, be there”, survive, competent, stay surviving, being the cause to the disaster, carry hope, endure,, recapturing life if possible, there´s not point of looking back, afraid	want to/have the power to send the partner away, be and stay healthy, not sick, doesn´t compete with their partner about being the sickest one, hope that you will survive your partner,	alone in a relationship and to people in general and to professionals, their partner doesn’t recognize them, enters a whole new world, are a stranger and at the same time married to a stranger, doesn’t want to be a stranger in the future	be quiet, knowing/missing/grief that you lost your partner, alone, their partner doesn’t express awareness about their existents as a person, be the one that doesn’t exist in the same way as before
